# Frequency and severity response scales for pain and discomfort: psychometric insights from EQ-HWB

**DOI:** 10.1007/s11136-025-04003-z

**Published:** 2025-06-10

**Authors:** Soumana C. Nasser, A. Simon Pickard, Jonathan L. Nazari, Maja Kuharic

**Affiliations:** 1 Department of Pharmacy Practice, School of Pharmacy, Lebanese America University, Byblos, Lebanon; 2 Department of Pharmacy Systems, Outcomes and Policy, Retzky College of Pharmacy, University of Illinois Chicago, Chicago, IL USA; 3 Department of Medical Social Sciences, Northwestern University Feinberg School of Medicine, 625 N Michigan Avenue, 21st floor, Chicago, IL 60603 USA

**Keywords:** Pain assessment, Health-related quality of life, Response scales, EQ-5D, EQHWB, Psychometric validation, CARE-2B

## Abstract

**Purpose:**

Pain and discomfort are core dimensions of health-related quality of life. This study aimed to compare and evaluate the psychometric properties of frequency versus severity response scales in assessing pain and discomfort and determining their unique measurement contributions across different health conditions.

**Methods:**

This secondary analysis utilized data from 1,008 participants derived through a dyadic study design of caregivers from the general population and their care recipients. Clinical data were based on self-reported health conditions. Pain and discomfort were assessed using the EQ-5D-5L and EQ-HWB experimental version. The analysis included Spearman's correlation, Shannon's indices, Item Response Theory (IRT), Differential Item Functioning (DIF), and ordinal logistic regression.

**Results:**

Pain frequency and severity items showed a strong correlation (r_s_=0.81, p<0.001), with similarly strong correlations across all pain and discomfort items (r_s_>0.5, p<0.001). Frequency scales demonstrated higher informativity than severity scales for both pain (H'=2.28, J'=0.98 vs H'=2.06, J'=0.89) and discomfort (H'=2.18, J'=0.94 vs H'=2.00, J'=0.86). IRT analysis revealed severity items had higher discrimination, while frequency items were more sensitive at lower trait levels. DIF analysis showed significant scale differences for pain (R2=0.24) but not discomfort (R2=0.001). Immunologic (OR=3.21) and musculoskeletal conditions (OR=2.65) were most strongly associated with pain frequency.

**Conclusion:**

Frequency and severity scales capture distinct aspects of pain and discomfort. Frequency scales provide better informativity across trait levels, while severity scales offer superior discrimination at higher intensities. For a comprehensive assessment, both scales are valuable; however, frequency scales may be preferable in shorter instruments due to their broader measurement range and higher informativity.

**Supplementary Information:**

The online version contains supplementary material available at 10.1007/s11136-025-04003-z.

## Background

Pain and discomfort are core dimensions of health-related quality of life (HRQoL) that present unique measurement challenges due to their subjective nature and complex manifestations [1, 2]. While pain and discomfort often occur together, they represent distinct experiences. Pain typically involves tissue damage and nociceptive responses [3], whereas discomfort is generally perceived as a broader, less intense, and more intermittent experience [1, 4]. Various factors influence how individuals perceive and report these symptoms, including the nature of the condition (acute vs. chronic), patient characteristics, and contextual elements [5, 6]. Research suggests that cultural, psychological, and social factors may also affect how individuals interpret and report pain and discomfort experiences [7, 8]. 

Assessing pain and discomfort in HRQoL patient-reported outcome measures (PROMs) involves various methodological considerations, particularly regarding the choice of response scale format and structure [9]. Existing generic HRQoL measures vary in their approaches to measuring pain and discomfort, with many instruments favoring severity scales. For instance, the EQ-5D uses a single item combining pain and discomfort with five severity levels [10]. The SF-36 includes two items related to pain: one to assess pain severity and another to measure pain interference with daily activities [11]. The PROMIS Global Health instrument uses a single item assessing average pain intensity on a 0–10 numerical rating scale [12, 13]. In contrast to these generic measures, condition-specific instruments often employ multiple items to capture the multifaceted nature of pain experience, addressing various dimensions such as intensity, frequency, and functional impact [8]. 

Despite these varying approaches, research in chronic pain suggests that both frequency and severity measures may provide valuable information. For instance, the Initiative on Methods, Measurement, and Pain Assessment in Clinical Trials (IMMPACT) recommends including both frequency and severity measures in pain assessment protocols for clinical trials [13]. However, the comparative advantages of these approaches in generic HRQoL instruments remain understudied, particularly regarding their measurement properties across different patient populations. The EQ Health and Wellbeing instrument (EQ-HWB), a recently developed instrument from the EuroQol Group that was in an experimental phase during this study period, presents a unique opportunity to examine these measurement questions [14–16]. This instrument includes a 25-item profile measure (EQ-HWB) and a shorter 9-item measure (EQ-HWB-S) [14–16]. 

Unlike other generic HRQoL instruments that typically use a single response scale for each symptom, the EQ-HWB incorporates both frequency and severity response scales for pain and discomfort items [14–16]. This design enables direct comparison of these measurement approaches within the same instrument and population, offering insights into their relative performance and utility. The present study seeks to evaluate the distinctiveness and psychometric performance of frequency versus severity response scales in assessing pain and discomfort within the EQ-HWB instrument. Specifically, we aim to analyze the relationship between frequency and severity responses, examine their measurement properties across different health conditions, and assess their relative informativity and discriminatory power. This research enhances our understanding of pain and discomfort measurement in HRQoL instruments and offers empirical evidence to guide response scale selection in future instrument development.

## Methods

### Study design and participants

This secondary analysis utilized data from a cross-sectional survey of 1,008 participants, aged 18 years or older, conducted between August 2022 and February 2023. The sample, derived through a dyadic study design, comprised 504 caregivers and their corresponding 504 care recipients. Caregivers were self-identified individuals from the general population who provided unpaid care to an adult relative or friend for at least six months. Care recipients were required to confirm they had received care from their caregiver. Detailed information about the study design and data collection methods has been previously published [17–19]. While the data source is dyadic, this study focuses on individual-level psychometric properties rather than caregiver and care recipient differences, allowing us to evaluate scale performance across a diverse range of health conditions. The University of Illinois Chicago Institutional Review Board approved the research (#2022 − 0490), and all participants signed informed consent.

### Data collection

Participants were recruited through the Qualtrics platform (Provo, UT, USA) using a caregiver panel. The sequential linking method was adopted, allowing dyads to complete the survey in one session without discussing their responses [20]. Caregivers provided informed consent, confirmed eligibility, and supplied details about their caregiving responsibilities. The survey included various validated instruments measuring health-related quality of life (EQ-5D-5L, EQ-HWB), wellbeing, and caregiving experiences (including CarerQoL, CARE-2B and other caregiver burden measures), all presented in randomized order to minimize sequencing effects. Quality control measures included attention checks and minimum completion time thresholds to ensure data integrity. For this specific analysis, we focus exclusively on the pain and discomfort dimensions from the EQ-5D-5L and EQ-HWB instruments. After completing the measures, participants answered demographic, clinical, and socioeconomic questions. Clinical information included self-reported chronic health conditions from a predefined list of conditions. Care recipients completed a similar sequence after caregivers finished their portion.

## Measures

The study implemented two measures assessing pain and discomfort: the EQ-5D-5L and EQ-HWB.

**EQ-5D-5 L** is a widely used preference-based measure designed to assess an individual’s HRQoL, with a recall period “today”, over five dimensions: mobility, self-care, usual activities, pain/discomfort, and anxiety/depression [10] Each dimension has five response levels: “no problems,” “slight problems,” “moderate problems,” “severe problems,” and “extreme problems/unable to perform.” The pain/discomfort dimension is a composite item that assesses both concepts together on a single severity scale. The EQ-5D-5L is supplemented by the Visual Analog Scale (EQ VAS) for assessing self-rated health on a scale from 0 (worst imaginable health) to 100 (best imaginable health).

**EQ-HWB/EQ-HWB-S** are instruments that include two measures: a 25-item profile measure (EQ-HWB) and a shorter 9-item classifier (EQ-HWB-S) embedded within the long measure, with a recall period “in the last 7 days” [14–16]. The EQ-HWB encompasses seven domains: activity, relationships, cognition, self-identity, autonomy, feelings, and physical sensations. Each item has five levels of frequency, severity, or difficulty. What makes the EQ-HWB distinctive is its approach to pain and discomfort assessment. The instrument includes four distinct items on physical pain and physical discomfort: (1) pain frequency, (2) pain severity, (3) discomfort frequency, and (4) discomfort severity. For the frequency items, the instrument uses a five-point scale: “none of the time,” “only occasionally,” “sometimes,” “often,” and “most or all of the time”. For the severity items, it uses a five-point scale: “no,” “mild,” “moderate,” “severe,” and “very severe.” At the time of this study, the EQ-HWB and EQ-HWB-S were in an experimental phase, and the EQ-HWB-S was in the process of finalization for distribution and licensing by the EuroQol Group. Among the items that may be further modified, the pain and discomfort items that are the focus of this investigation are not under consideration.

### Analysis

Data analysis included descriptive statistics of the respondent characteristics and the responses to the EQ-HWB and EQ-5D-5 L instruments. Continuous variables were reported as means and standard deviations (SD), and frequencies and proportions were used for categorical variables. All analyses were conducted using SAS Version 9.4 (SAS Institute Inc., Cary, NC, USA) and RStudio 2023.03.0 [21]. 

The psychometric properties of pain and discomfort frequency and severity scales were evaluated through five complementary analyses:

Correlational analysis examined relationships between response scales using Spearman rank-order coefficients to compare EQ-HWB physical pain severity and frequency, EQ-HWB physical discomfort severity and frequency, and EQ-5D physical pain/discomfort scales. Correlation strength (rₛ) was interpreted following Cohen’s guidelines: strong (> 0.50), moderate (0.31–0.50), weak (0.11–0.30), and trivial/none (< 0.10) [22]. These analyses were conducted for the full sample and subgroups by self-reported health conditions. The EQ-5D was included to determine whether these items capture overlapping constructs or distinct aspects of pain and discomfort. We expected all of the items related to physical discomfort and pain (severity and frequency) to be strongly correlated (*r* > 0.50) with each other.

Shannon’s Indices characterized scale informativity [23]. Shannon’s Index (H’) measured absolute informativity, while Shannon’s evenness index (J’) measured relative informativity or descriptive efficiency, controlling for response category count. Shannon’s Index (H’) is defined as H’ =–∑(n_i/N × log₂(n_i/N)), where C is the total number of response categories, n_i is the number of responses at the ith response category, and N is the total number of responses across all response categories. Thus, a greater H’ represents a greater amount of information captured by the descriptive system. Shannon’s Evenness Index (J’) is defined as J’ = H’/H‘(max), where H‘(max) = log₂(L). Higher values indicated greater informativity and discriminatory power, and J’ has a maximum value of 1.0 when there are the same number of responses per category. These indices were computed for the full sample and across sociodemographic and health condition subgroups, with informativity comparisons based on percent differences. Since all items had five response levels– and thus the same value for H‘(max)– we report only J’ values in our results, as they provide sufficient information for comparing informativity across scales. These indices were computed for the full sample and across sociodemographic and health condition subgroups, with informativity comparisons based on percent differences. We hypothesized that frequency scales would provide higher discriminatory power for physical pain and discomfort.

Item Response Theory analysis employed a graded response model (GRM) to analyze the EQ-HWB and EQ-5D physical pain/discomfort items [24]. A model incorporating all EQ-HWB and EQ-5D pain/discomfort items enabled a common reference point to identify the underlying construct. For each item, we estimated one discrimination parameter (a) indicating differentiation ability between trait levels, and four threshold parameters (d1-d4) representing trait levels at which respondents had 50% probability of endorsing each response category or higher [24]. Item characteristic curves (ICCs) were examined to visualize the probability of endorsing each response category across the latent trait continuum. Item information functions (IIFs) were analyzed to assess the precision of measurement provided by each item at different latent trait levels [24]. The analysis was conducted using the ‘mirt’ package (version 1.36.1) in RStudio 2023.03.0 [25]. We hypothesized that the frequency items would demonstrate higher informativity compared to severity for both pain and discomfort.

Differential item functioning (DIF) analysis was performed to assess whether respondents interpreted frequency and severity scales differently. The frequency scale was designated the referent group, and the severity scale as the focal group, rather than using traditional demographic or clinical subgroups for comparison. We evaluated uniform DIF, which occurs when a subgroup consistently responds to an item differently despite equal levels of the underlying trait. In contrast, non-uniform DIF arises when item responses vary across different levels of the underlying trait [26]. A hybrid logistic ordinal regression/IRT approach was used to examine uniform and non-uniform DIF. Three models were developed: [26] Model 1 (baseline): Included only the trait level (theta, θ); Model 2: Investigated uniform DIF by including scale type (frequency or severity) as a predictor; Model 3: Explored non-uniform DIF by including an interaction between trait level and scale type. DIF magnitude was evaluated using changes in McFadden’s pseudo-R², following guidelines which considered negligible if ΔR² < 0.02, moderate if 0.02 ≤ ΔR² < 0.13, and large if ΔR² ≥ 0.13 [27]. The analysis used the lordif R package, version 0.3-3 [28]. We anticipated distinct response patterns between severity and frequency scales when measuring physical pain and/or discomfort.

Finally, ordinal logistic regression models were implemented to examine relationships between demographic factors (age, gender), chronic health conditions, and the frequency and severity of physical pain and discomfort [29]. Separate models were constructed for each of the four EQ-HWB physical pain and discomfort items as dependent variables, with odds ratios (ORs) and 95% confidence intervals being reported. We expected that the frequency scale would receive higher endorsement across most health conditions.

## Results

Among the 1,008 participants, the majority were female (55%, *n* = 554), and the largest age group was 65 years and older (39.9%, *n* = 402). Approximately one-third of participants were employed (36.6%, *n* = 369), while the rest were retired or homemakers (36.8%, *n* = 371), unemployed or students (26.6%, *n* = 268). The most prevalent chronic health conditions were identified as hypertension (41.0%, *n* = 413), anxiety (36.3%, *n* = 366), and depression (33.2%, *n* = 335), followed by high cholesterol (29.9%, *n* = 301), diabetes (25.0%, *n* = 252), and gastrointestinal issues (22.3%, *n* = 225). A minority of participants (12.6%, *n* = 127) reported no health conditions (Table 1).

**Table 1:** Respondents characteristics and health conditions (*N* = 1,008)

Characteristics	*n* (%)		Health Conditions	*n* (%)
Gender				
Male	451 (44.7)		Heart	140 (13.9)
Female	554 (55.0)		Hypertension	413 (41)
Other	3 (0.3)		High cholesterol	301 (29.9)
Age (years)			Lung	103 (10.2)
18–44	328 (32.5)		Diabetes	252 (25)
45–64	278 (27.6)		Cancer	78 (7.7)
65+	402 (39.9)		Skin cancer	25 (2.5)
Race/ Ethnicity			Depression	335 (33.2)
White	731 (72.5)		Anxiety	366 (36.3)
Black or African American	158 (15.7)		Gastrointestinal	225 (22.3)
Asian	53 (5.3)		Musculoskeletal	221 (21.9)
Hispanic or Latino or Spanish Origin of any race	117 (11.6)		Ear, Eye, Nose, Throat	167 (16.6)
American Indian or Alaskan Native/ Other race	25 (2.5)		Neurologic	128 (12.7)
Marital status			Immunologic	75 (7.4)
Married, Living with a partner, or Engaged	598 (59.3)		Dermatologic	75 (7.4)
Widowed, Divorced or Separated	255 (25.3)		Endocrinologic	55 (5.5)
Single, never married	155 (15.4)		Chronic kidney disease	61 (6.1)
Employment status			Liver disease	27 (2.7)
Employed	369 (36.6)		Blood disorders	32 (3.2)
Retired or homemaker	371 (36.8)		Genitourinary	25 (2.5)
Unemployed or student	268 (26.6)		AIDS/HIV	7 (0.7)
General health rating			Other physical health	132 (13.1)
Excellent or Very good	239 (23.7)		Other mental health	72 (7.1)
Good	304 (30.2)		No health condition	127 (12.6)
Fair to Poor	465 (46.1)			

In the entire cohort and across chronic condition subgroups, strong correlations (rₛ ≥ 0.5, *p* < 0.001) were observed consistently among all four EQ-HWB items (pain frequency, pain severity, discomfort frequency, discomfort severity) and between each item and the EQ-5D pain/discomfort composite item. In the entire group, EQ-HWB pain severity exhibited the strongest correlation with both EQ-HWB pain frequency (rₛ= 0.81) and discomfort severity (rₛ= 0.81), followed by its correlation with the EQ-5D pain/discomfort composite item (rₛ= 0.76) and EQ-HWB discomfort frequency (rₛ= 0.62). The weakest correlation, though still strong, was between EQ-HWB discomfort frequency and the EQ-5D pain/discomfort composite item (rₛ= 0.58) (Table 2). Additionally, strong correlations were maintained across chronic condition subgroups with values above 0.70 between EQ-HWB pain severity and both EQ-HWB pain frequency and EQ-HWB discomfort severity (Table S1).

**Table 2:** Correlations between pain and discomfort items on EQ-HWB and EQ-5D-5 L (*N* = 1,008)

	EQ-HWB Pain Frequency	EQ-HWB Pain Severity	EQ-HWB Discomfort Frequency	EQ-HWB Discomfort Severity	EQ-5D Pain/ Discomfort
EQ-HWB Pain Frequency	1				
EQ-HWB Pain Severity	0.81	1			
EQ-HWB Discomfort Frequency	0.65	0.62	1		
EQ-HWB Discomfort Severity	0.73	0.81	0.71	1	
EQ-5D Pain/Discomfort	0.74	0.76	0.58	0.71	1

Note: All correlations were calculated using Spearman’s rank correlation coefficient (rₛ) and were statistically significant at *p* < 0.001. Empty cells indicate redundant correlations in the correlation matrix

Shannon’s Evenness Index (J’) showed that frequency scales demonstrated higher informativity compared to severity scales across both pain (J’=0.98 vs. J’=0.89) and discomfort dimensions (J’=0.94 vs. J’=0.86). This pattern was consistently observed across the majority of subgroups. The most pronounced differences in informativity between frequency and severity scales were observed for discomfort measurement in specific condition subgroups, with the highest percent differences being identified in dermatological (18.91%), gastrointestinal (17.39%), and neurologic conditions (16.63%). Conversely, the smallest differences were noted among participants reporting no health conditions, with minimal differences observed for both pain (2.62%) and discomfort (2.20%). When considering gender, age, or employment status, informativity remained higher for frequency versus severity across both pain and discomfort dimensions (Table 3).

**Table 3:** Shannon’s indices for pain and discomfort response scales across health conditions

	Pain Frequency	Pain Severity	Relative Informativity Frequency/ Severity	Discomfort Frequency	Discomfort Severity	Relative Informativity Frequency/ Severity
	J’ (f)	J’ (s)	f/s %**	J’ (f)	J’ (s)	f/s %
Entire sample	0.98	0.89	10.14%	0.94	0.86	8.61%
Heart	0.94	0.88	7.09%	0.94	0.87	7.62%
Hypertension	0.96	0.88	9.39%	0.96	0.88	8.45%
Cholesterol	0.98	0.91	7.31%	0.96	0.89	7.89%
Lung	0.87	0.88	-0.99%	0.98	0.87	12.15%
Diabetes	0.96	0.89	7.93%	0.96	0.87	9.88%
Cancer	0.92	0.93	-0.93%	0.98	0.93	5.43%
Depression	0.95	0.89	6.54%	0.97	0.87	9.84%
Anxiety	0.95	0.89	7.03%	0.97	0.88	9.35%
Gastrointestinal	0.91	0.83	9.38%	0.97	0.82	17.39%
Musculoskeletal	0.89	0.81	9.57%	0.97	0.83	15.75%
EENT	0.94	0.83	12.62%	0.98	0.84	15.17%
Immunologic	0.78	0.86	-10.03%	0.97	0.84	14.80%
Dermatologic	0.88	0.82	7.59%	0.95	0.78	18.91%
Neurologic	0.92	0.87	5.74%	0.98	0.83	16.63%
Other physical health	0.95	0.92	4.14%	0.99	0.89	11.01%
No health conditions	0.83	0.81	2.62%	0.79	0.78	2.20%

J’ = Shannon’s Evenness Index; f = frequency; s = severity; (f/s) = Ratio of J’ for frequency/severity. EENT = Eye, Ear, Nose, and Throat

*Higher values of J’ indicate greater informativity and discriminatory power of the scale

**For the relative informativity percentage, a positive value means in favor of frequency, and a negative value means in favor of severity

The IRT analysis revealed distinct patterns in difficulty parameters between severity and frequency scales. The EQ-HWB pain severity scale showed the highest discrimination parameter (a = 6.23) and the widest range of threshold parameters (d1 = 7.0 to d4=-10.33), suggesting strong differentiation across the pain continuum. In comparison, the EQ-HWB pain frequency scale showed moderate discrimination (a = 3.86) with a narrower threshold range (d1 = 4.61 to d4=-4.06). Similarly, the EQ-HWB discomfort severity scale exhibited good discrimination (a = 4.45) with a wide threshold range (d1 = 5.26 to d4=-8.40), while the EQ-HWB discomfort frequency scale had the lowest discrimination (a = 2.24) and the narrowest threshold range (d1 = 2.04 to d4=-4.25). (Table 4).

**Table 4:** Item response theory parameters for pain and discomfort items

	a1	d1	d2	d3	d4
EQ-HWB Pain Severity	6.23	7.00	0.62	-5.60	-10.33
EQ-HWB Pain Frequency	3.86	4.61	1.27	-1.55	-4.06
EQ-HWB Discomfort Severity	4.45	5.26	-0.24	-4.51	-8.40
EQ-HWB Discomfort Frequency	2.24	2.04	-0.19	-2.15	-4.25
EQ-5D Pain/Discomfort	3.37	2.66	-0.49	-3.58	-6.57

a = difficulty parameters and d = threshold parameters

The DIF analysis revealed substantial differences between frequency and severity scales for pain items but not for discomfort items. Large uniform DIF was observed for pain (ΔR²= 0.22), with negligible non-uniform DIF (ΔR²= 0.02), indicating that respondents systematically interpreted and responded to the pain frequency scale differently from the pain severity scale, regardless of their underlying level of pain. The total DIF for the pain item was large (ΔR²= 0.24). In contrast, both uniform and non-uniform DIF were negligible for discomfort items (ΔR²= 0.001 and ΔR²= 0.00, respectively). The total DIF for the discomfort item was negligible (ΔR²= 0.0013) **(**Table 5).

**Table 5:** Differential item functioning analysis of frequency severity response scales

	McFadden Pseudo ΔR² (Models 1 and 2)	McFadden Pseudo ΔR² (Models 2 and 3)	McFadden Pseudo ΔR² (Models 1 and 3)
Pain	0.224	0.019	0.243
Discomfort	0.001	0.000	0.001

Note: Values represent changes in McFadden’s pseudo R² between nested models, with EQ HWB severity scales as the reference group and EQ HWB frequency scales as the focal group. Model 1 includes only trait level; Model 2 adds scale type (uniform DIF); Model 3 includes trait level × scale type interaction (non-uniform DIF). DIF magnitude interpretation: ΔR² < 0.02 = negligible, 0.02 ≤ ΔR² < 0.13 = moderate, ΔR² ≥ 0.13 = large DIF

In ordinal logistic regression analyses, significantly higher odds of reporting both frequency and severity of pain and discomfort were observed among individuals with lung, diabetes, gastrointestinal, musculoskeletal, anxiety, or immunologic conditions compared to those without such conditions (*p* < 0.001). The highest odds of reporting pain frequency were observed among individuals with immunologic (OR 3.21, 95% CI: 1.97–5.24) or musculoskeletal conditions (OR 2.65, 95% CI: 1.95–3.61). Notably, depression was associated with higher odds of reporting pain frequency (OR 1.51, 95% CI: 1.08–2.11) and discomfort frequency (OR 1.50, 95% CI: 1.08–2.09), but not their severity. Cancer (OR 1.84, 95% CI: 1.17–2.89) and dermatologic conditions (OR = 1.61, 95% CI: 1.02–2.55) were significantly associated with increased pain frequency, but not with pain severity or discomfort frequency/severity. In contrast, neurological conditions were significantly associated with pain severity (OR 1.47, 95% CI: 1.01–2.13) but not with pain frequency or discomfort frequency/severity. Heart conditions were significantly associated only with discomfort frequency (OR 1.62, 95% CI: 1.13–2.31). Regarding demographic factors, females were less likely to report pain severity compared to males (OR 0.71, 95% CI 0.56–0.90) (Table 6).

**Table 6:** Associations between health conditions and pain/discomfort response scales

	EQ-HWB Pain Frequency	EQ-HWB Pain Severity	EQ-HWB Discomfort Frequency	EQ-HWB Discomfort Severity
	OR [95% CI]	OR [95% CI]	OR [95% CI]	OR [95% CI]
Age Category				
(18–44 y.o.) vs. (65 + y.o.)	1.35 [0.90, 2.00]	1.55 [1.03, 2.32] *	1.27 [0.85, 1.88]	1.56 [1.04, 2.34] *
(45–64 y.o.) vs. (65 + y.o.)	1.30 [0.91, 1.87]	1.31 [0.91, 1.88]	1.33 [0.93, 1.89]	1.29 [0.89, 1.85]
Gender (female vs. male)	0.87 [0.69, 1.11]	0.71 [0.56, 0.90]	0.92 [0.73, 1.17]	0.87 [0.68, 1.11]
Health Conditions				
Heart	1.24 [0.86, 1.78]	1.19 [0.83, 1.72]	1.62 [1.13, 2.31] *	1.38 [0.96, 1.99]
Hypertension	1.22 [0.93, 1.60]	1.21 [0.92, 1.59]	1.13 [0.86, 1.47]	1.21 [0.92, 1.59]
High cholesterol	0.91 [0.69, 1.22]	0.80 [0.60, 1.06]	0.88 [0.66, 1.17]	0.80 [0.59, 1.06]
Lung	2.04 [1.36, 3.06]*	1.67 [1.12, 2.49] *	1.54 [1.04, 2.27] *	1.60 [1.08, 2.39] *
Diabetes	1.67 [1.26, 2.22] *	1.64 [1.23, 2.18] *	1.63 [1.23, 2.15] *	1.61 [1.21, 2.15] *
Cancer	1.84 [1.17, 2.89] *	1.47 [0.94, 2.31]	1.33 [0.86, 2.07]	1.51 [0.96, 2.37]
Depression	1.51 [1.08, 2.11] *	1.20 [0.86, 1.68]	1.50 [1.08, 2.09] *	1.19 [0.85, 1.66]
Anxiety	1.41 [1.02, 1.96] *	1.45 [1.04, 2.01] *	1.50 [1.08, 2.07] *	1.61 [1.15, 2.24] *
Gastrointestinal	1.79 [1.32, 2.42] *	1.56 [1.15, 2.12] *	1.51 [1.12, 2.04] *	1.52 [1.12, 2.07] *
Musculoskeletal	2.65 [1.95, 3.61] *	1.63 [1.20, 2.21] *	1.40 [1.04, 1.89] *	1.38 [1.01, 1.87] *
Eye, Ear, Nose, and Throat	0.92 [0.66, 1.29]	0.83 [0.59, 1.17]	1.07 [0.77, 1.50]	0.97 [0.69, 1.37]
Immunologic	3.21 [1.97, 5.24] *	2.10 [1.32, 3.34] *	1.45 [0.92, 2.28]	1.60 [1.01, 2.53] *
Dermatologic	1.61 [1.02, 2.55] *	1.04 [0.66, 1.63]	1.40 [0.90, 2.19]	1.01 [0.64, 1.59]
Neurologic	1.03 [0.71, 1.49]	1.47 [1.01, 2.13] *	1.14 [0.79, 1.65]	1.29 [0.89, 1.87]
Other physical health	1.54 [1.09, 2.19] *	1.47 [1.04, 2.09] *	1.38 [0.98, 1.95]	1.48 [1.04, 2.10] *
No health conditions	0.68 [0.45, 1.02]	0.71 [0.47, 1.07]	0.86 [0.57, 1.29]	0.71 [0.47, 1.09]

Note: Values represent odds ratios with 95% confidence intervals in brackets. Reference categories: Age (65+ years), Gender (male), Health conditions (absence of condition). *p < 0.05

## Discussion

The present study evaluated the distinctiveness and unique contributions of frequency versus severity response scales in assessing pain and discomfort using the EQ-HWB/EQ-HWB-S instrument. Our analysis revealed four key findings: (1) strong correlations between pain and discomfort measures, (2) higher informativity of frequency scales across conditions, (3) better discrimination of severity scales at higher trait levels, and (4) differential item functioning between frequency and severity scales for pain but not discomfort. These insights advance our understanding of optimal approaches to health measurement scales.

The strong associations found between all EQ-HWB physical pain and discomfort items align with previous research, suggesting that pain and discomfort are closely related constructs that frequently co-occur [30, 31]. Despite these correlations, our findings indicate that frequency and severity scales may capture distinct aspects of pain and discomfort experiences, providing complementary information for health assessment. Our correlation analyses revealed stronger relationships between the EQ-5D-5L pain/discomfort composite item and the EQ-HWB pain measures compared to its relationships with discomfort measures. These findings align with Engel et al.‘s (2023) observation that the EQ-5D-5L pain/discomfort dimension predominantly captures pain rather than discomfort [31]. Our work extends their research through comprehensive psychometric analyses while providing quantitative evidence that supports their qualitative findings.

The combined use of IRT, DIF, and Shannon’s indices revealed complementary insights about scale functioning. The IRT analysis revealed important differences in how frequency and severity scales function across the trait continuum. Severity scales demonstrated higher discrimination parameters and wider threshold ranges, indicating better measurement precision at higher trait levels. In contrast, frequency scales with their narrower threshold ranges provided greater sensitivity for detecting and differentiating milder to moderate pain and discomfort experiences. Shannon’s indices complemented these findings by demonstrating superior informativity for frequency scales across the measurement continuum, reflecting more efficient utilization of response categories. These complementary findings highlight the different strengths of each scale type. IRT parameters reveal that severity scales excel at distinguishing between levels of high-intensity symptoms, making them potentially more valuable for clinical populations with more severe conditions. Conversely, the superior informativity of frequency scales shown by Shannon’s indices suggests they distribute information more evenly across response categories, making them particularly suitable for general population assessment and early detection. Using our novel DIF analysis approach with frequency and severity scales as comparison groups, we found that respondents interpreted and responded differently to pain frequency versus pain severity scales, but showed negligible differences for discomfort scales. This suggests that pain frequency and severity scales may measure distinct aspects of the pain experience, whereas discomfort frequency and severity scales may capture a more uniform understanding of discomfort. This distinction has not been previously documented in HRQoL measurement literature and provides new insights into how respondents understand and report these constructs.

The findings of this study have important implications for assessing pain and discomfort in HRQoL instruments. When developing or selecting HRQoL instruments, researchers and clinicians should consider the relative merits of frequency and severity scales based on the specific goals of the assessment, for longer instruments, including both frequency and severity scales, may offer complementary information about different aspects of pain and discomfort experiences. However, for a shorter instrument (e.g., EQ-HWB-S), careful consideration of response scale properties is essential, with our findings indicating that frequency scales offer advantages in informativity across a broader range of pain and discomfort experiences. While different response scales may be optimal for different conditions, implementing condition-specific scales in preference-based instruments like the EQ-5D and EQ-HWB-S would require multiple value sets, increasing complexity and compromising cross-condition comparability in utility measurement for quality-adjusted life years (QALYs). This practical constraint supports using a single response scale type across conditions. Additionally, severity scales may offer benefits in valuation procedures and international applications that extend beyond the psychometric properties examined in this study.

Our ordinal logistic regression analysis revealed distinct patterns across different conditions, suggesting that optimal measurement approaches may need to be condition-specific. In conditions such as immunologic disorders, musculoskeletal conditions, depression, cancer, and dermatologic conditions, frequency scales demonstrated particular value in capturing the temporal nature of symptom experiences. Conversely, severity scales provided more meaningful information in neurological conditions where symptom intensity often characterizes the experience. The choice of recall period is particularly important for frequency scales, as it needs to be long enough to capture meaningful patterns of symptom occurrence while remaining short enough for accurate recall. The EQ-HWB’s 7-day period appears to work well for frequency measurement across conditions, while the EQ-5D’s ‘today’ recall period, though minimizing recall bias, may be less suitable for frequency measurement, particularly in conditions with fluctuating symptoms. Future research should systematically examine how different recall periods affect frequency versus severity scale performance.

Several limitations should be noted when interpreting the findings of this study. Our cross-sectional data preclude causal inferences about the relationships between health conditions and pain/ discomfort experiences. Relying on self-reported health conditions may have particular subjectivity, as some participants may be unaware of their diagnosis or unintentionally misreport them. While our sample included diverse health conditions, the generalizability of these findings to different cultural and linguistic contexts requires further validation through replication studies.

## Conclusion

This study demonstrated the complementary nature of frequency and severity scales in assessing physical pain and discomfort. Frequency scales show higher informativity across the response options and greater sensitivity at lower levels of the trait, while severity scales provide better discrimination at higher levels of the trait. For a longer instrument, incorporating both scale types offers complementary information about different aspects of pain and discomfort experiences. However, for a shorter instrument, the frequency scale may be preferred due to its higher informativity and ability to capture a broader range of pain and discomfort traits. The selection of frequency or severity scales, or a combination of both, should be guided by the specific goals of the assessment, the target population, and the instrument’s intended use. Future research should continue to explore the optimal approaches to assessing pain and discomfort across different clinical populations and measurement contexts.

## Electronic supplementary material

Below is the link to the electronic supplementary material.

Supplementary Material 1

Supplementary Material 2

## Data Availability

The data that support the findings of this study are available from the corresponding author upon reasonable request.
