# Supplementary material for: Frequency and severity response scales for pain and discomfort: psychometric insights from EQ-HWB
Source: Qual Life Res. 2025 Jun 10;34(8):2259–68. doi: 10.1007/s11136-025-04003-z (PMC12274242; doi:10.1007/s11136-025-04003-z)
Supplement: Supplementary file 1 — Supplementary Material 1 [file 11136_2025_4003_MOESM1_ESM.docx]

**Table S1.** Correlation Between Pain and Discomfort, Frequency and Severity Within EQ-HWB-25 in Subgroup*s*

| Subgroups of Health Conditions |  | EQ-HWB  Pain Frequency | EQ-HWB  Pain Severity | EQ-HWB  Discomfort Frequency | EQ-HWB Discomfort Severity |
| --- | --- | --- | --- | --- | --- |
| Anxiety | EQ-HWB Pain Frequency | 1 | 0.76 | 0.63 | 0.65 |
|  | EQ-HWB Pain Severity |  | 1 | 0.62 | 0.77 |
|  | EQ-HWB Discomfort Frequency |  |  | 1 | 0.74 |
|  | EQ-HWB Discomfort Severity |  |  |  | 1 |
| Depression | EQ-HWB Pain Frequency | 1 | 0.75 | 0.60 | 0.66 |
|  | EQ-HWB Pain Severity |  | 1 | 0.59 | 0.79 |
|  | EQ-HWB Discomfort Frequency |  |  | 1 | 0.70 |
|  | EQ-HWB Discomfort Severity |  |  |  | 1 |
| Heart | EQ-HWB Pain Frequency | 1 | 0.79 | 0.62 | 0.75 |
|  | EQ-HWB Pain Severity |  | 1 | 0.56 | 0.74 |
|  | EQ-HWB Discomfort Frequency |  |  | 1 | 0.70 |
|  | EQ-HWB Discomfort Severity |  |  |  | 1 |
| Musculoskeletal | EQ-HWB Pain Frequency | 1 | 0.80 | 0.52 | 0.70 |
|  | EQ-HWB Pain Severity |  | 1 | 0.54 | 0.77 |
|  | EQ-HWB Discomfort Frequency |  |  | 1 | 0.65 |
|  | EQ-HWB Discomfort Severity |  |  |  | 1 |
| Neurologic | EQ-HWB Pain Frequency | 1 | 0.72 | 0.64 | 0.61 |
|  | EQ-HWB Pain Severity |  | 1 | 0.49 | 0.73 |
|  | EQ-HWB Discomfort Frequency |  |  | 1 | 0.62 |
|  | EQ-HWB Discomfort Severity |  |  |  | 1 |
| Lung | EQ-HWB Pain Frequency | 1 | 0.77 | 0.60 | 0.65 |
|  | EQ-HWB Pain Severity |  | 1 | 0.57 | 0.78 |
|  | EQ-HWB Discomfort Frequency |  |  | 1 | 0.73 |
|  | EQ-HWB Discomfort Severity |  |  |  | 1 |
| Cancer | EQ-HWB Pain Frequency | 1 | 0.78 | 0.71 | 0.68 |
|  | EQ-HWB Pain Severity |  | 1 | 0.65 | 0.80 |
|  | EQ-HWB Discomfort Frequency |  |  | 1 | 0.70 |
|  | EQ-HWB Discomfort Severity |  |  |  | 1 |
| Immunologic | EQ-HWB Pain Frequency | 1 | 0.74 | 0.45 | 0.59 |
|  | EQ-HWB Pain Severity |  | 1 | 0.41 | 0.74 |
|  | EQ-HWB Discomfort Frequency |  |  | 1 | 0.62 |
|  | EQ-HWB Discomfort Severity |  |  |  | 1 |
| Diabetes | EQ-HWB Pain Frequency | 1 | 0.73 | 0.66 | 0.68 |
|  | EQ-HWB Pain Severity |  | 1 | 0.60 | 0.79 |
|  | EQ-HWB Discomfort Frequency |  |  | 1 | 0.71 |
|  | EQ-HWB Discomfort Severity |  |  |  | 1 |
| Hypertension | EQ-HWB Pain Frequency | 1 | 0.77 | 0.60 | 0.70 |
|  | EQ-HWB Pain Severity |  | 1 | 0.58 | 0.79 |
|  | EQ-HWB Discomfort Frequency |  |  | 1 | 0.70 |
|  | EQ-HWB Discomfort Severity |  |  |  | 1 |
| Ear, Eye, Nose, Throat | EQ-HWB Pain Frequency | 1 | 0.81 | 0.50 | 0.67 |
|  | EQ-HWB Pain Severity |  | 1 | 0.58 | 0.80 |
|  | EQ-HWB Discomfort Frequency |  |  | 1 | 0.71 |
|  | EQ-HWB Discomfort Severity |  |  |  | 1 |
| Gastrointestinal | EQ-HWB Pain Frequency | 1 | 0.74 | 0.61 | 0.61 |
|  | EQ-HWB Pain Severity |  | 1 | 0.56 | 0.73 |
|  | EQ-HWB Discomfort Frequency |  |  | 1 | 0.69 |
|  | EQ-HWB Discomfort Severity |  |  |  | 1 |
| No Health Conditions | EQ-HWB Pain Frequency | 1 | 0.72 | 0.64 | 0.61 |
|  | EQ-HWB Pain Severity |  | 1 | 0.49 | 0.73 |
|  | EQ-HWB Discomfort Frequency |  |  | 1 | 0.62 |
|  | EQ-HWB Discomfort Severity |  |  |  | 1 |
| All p-values are significant at 0.05 | | | | | |
