# Supplementary material for: Frequency and severity response scales for pain and discomfort: psychometric insights from EQ-HWB
Source: Qual Life Res. 2025 Jun 10;34(8):2259–68. doi: 10.1007/s11136-025-04003-z (PMC12274242; doi:10.1007/s11136-025-04003-z)
Supplement: Supplementary file 2 — Supplementary Material 2 [file 11136_2025_4003_MOESM2_ESM.docx]

STROBE Statement—Checklist of items that should be included in reports of ***cross-sectional studies***

|  | Item No | Recommendation | Page No |
| --- | --- | --- | --- |
| **Title and abstract** | 1 | (*a*) Indicate the study’s design with a commonly used term in the title or the abstract | ✔ Yes – Abstract mentions "cross-sectional survey" |
|  |  | (*b*) Provide in the abstract an informative and balanced summary of what was done and what was found | ✔ Yes – Abstract summarizes objectives, methods, results |
| Introduction | | | |
| Background/rationale | 2 | Explain the scientific background and rationale for the investigation being reported | ✔ Yes – "Background" section (p.1–2) |
| Objectives | 3 | State specific objectives, including any prespecified hypotheses | ✔ Yes – End of Background, beginning of Methods (p.2) |
| Methods | | | |
| Study design | 4 | Present key elements of study design early in the paper | ✔ Yes – Start of Methods (p.2) |
| Setting | 5 | Describe the setting, locations, and relevant dates, including periods of recruitment, exposure, follow-up, and data collection | ✔ Yes – "Study Design and Participants" (p.2) |
| Participants | 6 | (*a*) Give the eligibility criteria, and the sources and methods of selection of participants | ✔ Yes – p.2 (caregiver/care recipient criteria) |
| Variables | 7 | Clearly define all outcomes, exposures, predictors, potential confounders, and effect modifiers. Give diagnostic criteria, if applicable | ✔ Yes – p.3–4 (pain/discomfort items, demographics) |
| Data sources/ measurement | 8* | For each variable of interest, give sources of data and details of methods of assessment (measurement). Describe comparability of assessment methods if there is more than one group | ✔ Yes – p.3 (measures section), p.4 (instruments) |
| Bias | 9 | Describe any efforts to address potential sources of bias | To address potential sources of bias, we randomized the order of measures to minimize sequence effects and implemented quality control measures including attention checks. The use of self-reported health conditions is acknowledged as a limitation that may introduce misclassification bias. |
| Study size | 10 | Explain how the study size was arrived at | Sample size is reported (p.2), of 1,008 participants was determined based on power calculations indicating this would provide sufficient statistical power (>80%, α=0.05) to detect meaningful differences in informativity and discrimination parameters between frequency and severity scales. |
| Quantitative variables | 11 | Explain how quantitative variables were handled in the analyses. If applicable, describe which groupings were chosen and why | ✔ Yes – p.4–6 (statistical analysis) |
| Statistical methods | 12 | (*a*) Describe all statistical methods, including those used to control for confounding | ✔ Yes – p.4–6 (IRT, regression, DIF) |
|  |  | (*b*) Describe any methods used to examine subgroups and interactions | ✔ Yes – Start of Methods (p.2) |
|  |  | (*c*) Explain how missing data were addressed | ✔ Yes – "Study Design and Participants" (p.2) |
|  |  | (*d*) If applicable, describe analytical methods taking account of sampling strategy | N/A |
|  |  | (*e*) Describe any sensitivity analyses | N/A |
| Results | | | |
| Participants | 13* | (a) Report numbers of individuals at each stage of study—eg numbers potentially eligible, examined for eligibility, confirmed eligible, included in the study, completing follow-up, and analysed | The study included 1,008 participants (504 caregiver-care recipient dyads) recruited through a Qualtrics panel. From an initial pool of 4,714 individuals who started the survey, 2,651 were excluded prior to eligibility screening. Of the remaining 2,063 participants who passed eligibility questions, 957 failed validity checks, and an additional 317 did not pass security and quality checks. At this stage, 789 participants remained. From this pool, 285 participants were excluded due to quota requirements for demographics (race, gender), resulting in 504 complete dyads that were included in the final analysis. All analyses were conducted using this complete dataset, with no excluded observations or loss to follow-up after enrollment. |
|  |  | (b) Give reasons for non-participation at each stage | ✔ Yes – p.6 (demographics, health conditions) |
|  |  | (c) Consider use of a flow diagram | A flow diagram of participant selection was included in a previous publication using the same dataset (Kuharic et al., 2024) and was not duplicated for this manuscript. |
| Descriptive data | 14* | (a) Give characteristics of study participants (eg demographic, clinical, social) and information on exposures and potential confounders | ✔ Yes – Results include detailed psychometric outcomes |
|  |  | (b) Indicate number of participants with missing data for each variable of interest | ✔ Yes – Odds ratios, IRT, Shannon indices (p.6–10) |
| Outcome data | 15* | Report numbers of outcome events or summary measures | ✔ Yes – DIF, IRT, subgroup analyses included |
| Main results | 16 | (*a*) Give unadjusted estimates and, if applicable, confounder-adjusted estimates and their precision (eg, 95% confidence interval). Make clear which confounders were adjusted for and why they were included | Unadjusted estimates are reported throughout the Results section, including correlation coefficients, Shannon's Indices, IRT parameters, and odds ratios with 95% confidence intervals for logistic regression analyses. No confounder-adjusted estimates were performed as the primary focus was on psychometric properties rather than causal associations. |
|  |  | (*b*) Report category boundaries when continuous variables were categorized | ✔ Yes – p.6 (demographics, health conditions) |
|  |  | (*c*) If relevant, consider translating estimates of relative risk into absolute risk for a meaningful time period | N/A |
| Other analyses | 17 | Report other analyses done—eg analyses of subgroups and interactions, and sensitivity analyses | ✔ Yes – Results include detailed psychometric outcomes |
| Discussion | | | |
| Key results | 18 | Summarise key results with reference to study objectives | ✔ Yes – p.10 |
| Limitations | 19 | Discuss limitations of the study, taking into account sources of potential bias or imprecision. Discuss both direction and magnitude of any potential bias | ✔ Yes – p.11 |
| Interpretation | 20 | Give a cautious overall interpretation of results considering objectives, limitations, multiplicity of analyses, results from similar studies, and other relevant evidence | ✔ Yes – p.11–12 |
| Generalisability | 21 | Discuss the generalisability (external validity) of the study results | ✔ Yes – p.11 |
| Other information | | | |
| Funding | 22 | Give the source of funding and the role of the funders for the present study and, if applicable, for the original study on which the present article is based | ✔ Yes – End of manuscript (EuroQol Group) |

*Give information separately for exposed and unexposed groups.

**Note:** An Explanation and Elaboration article discusses each checklist item and gives methodological background and published examples of transparent reporting. The STROBE checklist is best used in conjunction with this article (freely available on the Web sites of PLoS Medicine at http://www.plosmedicine.org/, Annals of Internal Medicine at http://www.annals.org/, and Epidemiology at http://www.epidem.com/). Information on the STROBE Initiative is available at www.strobe-statement.org.
